# The Cost of Provisions in Institutions.—XVI

**Published:** 1904-08-27

**Authors:** 


### HOSPITAL ADMINISTRATION.

### CONSTRUCTION AND ECONOMICS.

### THE COST OF PROVISIONS IN INSTITUTIONS.—XVI.

BY THE AUTHOR OF "HOSPITAL EXPENDITURE—THE COMMISSARIAT."

(*Continued from page* 366.)

CONCLUSION.

THE comparison attempted in a previous article between the cost per bed for provisions of some London hospitals went far to prove that a low average of expenditure resulted in most instances from a low average of persons "other than inmates" boarded in the institution. And this is exactly what might be expected. The tendency is more and more, however, to gather the workers within the walls for meals, the gain in time and wages far more than compensating for the extra expenditure on food. And hence the study for economy must go much deeper than mere limitation of numbers. To sum up—The first and surest strand in economy is a system of accounts which shall show the weekly variations in cost of boarding the different sections of the household. Of this we need say no more.

Second in importance is an intelligent method of inviting and accepting contracts. In too many hospitals this important duty is a matter of dull routine, none of the governors having the necessary zeal to master the question of prices and ascertain the best local sources of supply. The common practice of accepting contracts for the whole year is a great bar to a clear understanding of the right price to pay. Yearly contracts render it impossible at any moment to make comparisons with market prices, for in the plentiful season the contractor must recoup himself for losses incurred when he is supplying goods at a loss; and it is a matter of certainty that he will leave himself a wide margin on the right side. To ensure that the goods are supplied at the current market price the contract should not run for more than three months. Competition should be invited, and although it is important to guard against low tenders, framed with a view to cutting prices, the contrary error is perhaps the more common, of lazily placing blind confidence in firms which for years have charged their own prices, and have grown to regard the hospital as a comfortable source of revenue.

Third in order of importance in the quest for economy is a trained steward or housekeeper responsible for receiving the goods from the contractor. Considering the cost and quantity of the provisions which are consumed in even a moderate sized hospital, it is amazing that *training* in this official is the exception rather than the rule. Whoever thinks of subjecting an applicant for such a post to even the most elementary tests of his knowledge? He will have to superintend day by day the weighing of hundredweights of bread. Does he know by looking at it the difference between a slice of bread made with "seconds" and with the best flour? Ask the baker's boy and see what he will say to such a test. Year after year the steward of a big institution sees the meat brought in, and notes the weight. If its condition affect his nose he will make complaint. But can he detect that Australian beef has been substituted for American, or that the mutton is from the woolly ewes of Argentina instead of from Wellington or Canterbury? Ask the meat salesman's apprentice the same question, and he will smile as your steward would smile if he were asked whether he really knew the difference between Greek and Latin characters. The knowledge is accessible to all, but it is knowledge which very few persons outside the "trade" deem it worth their while to acquire. Again, if the Stores List be examined, four or five differently priced sugars will be found under the head of moist and loaf respectively. Is it not reasonable to expect that the person who receives the groceries shall be able to detect unerringly if an inferior quality be substituted in place of what is ordered? There is no mystery in these things. There is a corrrct price to pay for each quality, and the common practice of leaving the matter in the hands of the contractor and his subordinate is at the root of much of the bad catering justly complained of in institutions.

The fourth essential in economical housekeeping is good larder accommodation. It is hardly necessary to enlarge on this point, seeing that it is fully recognised in all modern buildings; but we believe that if the full significance of the larder were understood in connection with economy an effort would be made in all antiquated institutions to put the storage of perishable victuals on a better footing. The sterilisation of milk has obviated much waste in this direction, but it has not abolished the necessity for a cool larder for dairy produce. Separate storage for the bread and a good store-room fitted with drawers and bins for dry goods are also essential, and present no difficulties in practice. But a good meat larder is by no means easy of attainment, and many apparently ideal larders are found to fail on trial in just the one essential point, that of keeping the meat sweet. To be cold and dark, yet not dank, and to be freely ventilated with the right sort of air, is not within the reach of every town larder, but it will well repay the hospital to call in the best available skill to rectify any defect in the storage of the meat. Good larders will make it possible to buy the meat in bulk and thus reduce its price to the minimum; they will obviate all waste from the turning off of meat in hot weather, by which more expense is incurred than any one but the housekeeper fully realises; and, lastly, they will place it in the power of the cook, if she knows her business properly, to hang the meat a sufficient length of time to render it properly tender when cooked.

And this brings us to the fifth important strand in hospital economy—the cook. An institution cook is generally the product of the institution itself. She has begun in the hospital as scullery-maid, advanced to the learning of her business as kitchen-maid, and from that position rises to be mistress of the kitchen. She knows her business thoroughly, so far as the handling of big joints and the management of the apparatus goes, and she understands the peculiar routine of the meals. But, alas! it is to be feared she has had no teaching of the kind which is now available in many centres on the "true inwardness" of cooking. And it is to be feared that what she is at the outset of her career as full-blown cook that she remains to the end, for she may pass through countless institutions, little and big, and find no matron with either the time or the knowledge to teach her better. In a household where every inmate is working at high pressure, and must look to good digestion for ability to stand the strain of incessant hard work, the cook is the unseen pivot on whom the comfort of all depends. The demand for good institution cooks is great, and cannot be satisfactorily met by the promotion of untrained kitchen-maids. The cookery schools would do well, we think, to train women specially for this work, which is well suited to a superior class, for the head cook in a large institution is in a position of great responsibility with many subordinates. We believe that higher wages in this direction would result in securing better trained women and would lead to an immediate reduction of expenses, and that much of the extravagance perceptible in hospital housekeeping is due to the absurd parsimony of placing a cheap, untrained, ignorant woman at the head of the kitchen.

In conclusion, the writer would like to point out that in this, as in the preceding, articles no ounce of *theory* finds a place. The conclusions which have been set before the reader are based on actual facts, the experience of hospital managers who have devoted their lives to battling with farthings in the service of the poor. Economy is not generally considered an ennobling or heroic virtue, and directed to personal ends it may have a belittling tendency. But regarded as a high duty in the wise administration of public funds, it can dignify the humblest tasks, and, leavened by self-sacrifice, in the formation of character it acts a bracing part.

